# Identification of potential lncRNA‐miRNA‐mRNA regulatory network contributing to aldosterone‐producing adenoma

**DOI:** 10.1111/jcmm.17586

**Published:** 2022-10-27

**Authors:** Minghui Bao, Haotong Li, Jianping Li

**Affiliations:** ^1^ Department of Cardiology, Peking University First Hospital Peking University Beijing China; ^2^ National Center for Cardiovascular Diseases, Fuwai Hospital Chinese Academy of Medical Sciences and Peking Union Medical College Beijing China

**Keywords:** aldosterone‐producing adenoma, competing endogenous RNA, hub gene, hypertension, lncRNA, transcriptional analysis

## Abstract

Aldosterone‐producing adenoma (APA) is a common cause of secondary hypertension. This study aimed to explore the lncRNA‐miRNA‐mRNA competitive endogenous RNA (ceRNA) network to uncover molecular mechanism underlying APA. The mRNA and lncRNA expression data of APA and adjacent adrenal gland (AAG) from GSE60044, GSE64957 and GSE101894 were obtained from the Gene Expression Omnibus (GEO) database to analyse differentially expressed genes (DEGs) and lncRNAs (DElncs). Hub genes were identified by robust rank aggregation (RRA) and protein–protein interaction (PPI) network analysis. The miRcode and miRWalk network tools were used to construct the ceRNA network. 1526 upregulated and 1512 downregulated DEGs were identified, which are mainly enriched in extracellular matrix and Ca^2^^+^‐related GO terms. In the KEGG pathway analysis, Ca^2+^ signalling and the aldosterone synthesis and secretion pathways were enriched. ceRNA network included 2 lncRNAs, 9 miRNAs, and 13 mRNAs. The lncRNAs are MEG3 and LINC00115. The mRNAs included CCND1, TP53, GPRC5B, BMI1, COMMD3‐BMI1, ADAMTS15, STAT3, MMP2, SCN2B, CXCL12, HGF, FOS, and THBS1. Overall, this study conducted a ceRNA regulatory network analysis and identified that 2 lncRNAs and 13 mRNAs may contribute to the development of APA. These findings may provide novel diagnostic and intervention targets for APA.

## 1 INTRODUCTION

Primary aldosteronism (PA) is the most common cause of endocrine hypertension.^1^ It is a heterogeneous group of disorders characterized by hypertension and aldosterone overproduction relatively autonomous from the renin–angiotensin system. The prevalence of PA is around 5% in patients with hypertension in primary care^2^ and up to 20% in patients with resistant hypertension.^3^ Compared with patients having primary hypertension harbouring matched CV risk profiles, patients with PA are likely to have increased risk of CV and cerebrovascular events, as well as the increased rate of metabolic syndrome.^4^

Unilateral aldosterone‐producing adenoma (APA) is a major PA subtype. Somatic mutations drive autonomous aldosterone production in most adenomas. APA is underdiagnosed because it does not have a specific and easily identifiable feature. The diagnostic investigation is a multistep process of screening, confirmatory testing, and subtype differentiation of APA from bilateral forms for therapeutic management. Adrenal venous sampling is key for reliable subtype identification. For unilateral disease, surgery offers the possibility of cure, with total laparoscopic unilateral adrenalectomy being the treatment of choice, while bilateral disease is treated mainly with mineralocorticoid receptor antagonists. Prompt diagnosis and targeted treatment of APA can normalize both blood pressure and excessive aldosterone production, mitigate aldosterone‐specific target organ damage, improve quality of life, and reduce mortality. However, the diagnosis and treatment of APA are frequently limited by the complex diagnostic processes, the invasive treatment strategy, and the great medical expense.^5^ Consequently, understanding the potential pathogenic mechanisms of APA may contribute to the diagnosis and targeted treatment of this disease.

Although the aetiology of APA remained unclear, various studies have conducted gene expression profile analyses of APA compared with adjacent adrenal gland (AAG) or normal adrenals. The findings have expanded our understanding of the genetic and molecular mechanisms of PA. Recurrent pathogenic somatic mutations underlie the pathophysiology of most APA. Exome sequencing has identified mutations in ion channels‐related genes, such as CACNA1D, CACNA1H, ATP1A1, ATP2B3, KCNJ5, and CLCN2^6^, ^7^, ^8^, ^9^. Causative mutations of these genes lead to depolarization of the cell membrane and the depolarization could further activate voltage‐gated Ca^2+^ channels, promote the transcription of aldosterone synthase (CYP11B2). In addition, other APA pathogenic genes such as VSNL1, CALN1, GSTA1, NPNT, and CLGN have also been found to be involved in aldosterone overproduction.^10^, ^11^, ^12^, ^13^

Competing endogenous RNA (ceRNA), a new regulatory mechanism between coding RNA and noncoding RNA, is defined as RNA molecules compete for a common miRNA with other RNAs through miRNA response elements (MREs).^14^ Accumulating evidences have demonstrated that lncRNAs, like ceRNAs, can compete for MREs with diseases risk genes and further participate in the occurrence and development of neoplastic diseases.^15^ However, the potential role of ceRNAs in APA remains unclear. Considering the importance of these functional modules, there is a need to construct ceRNA network to explore lncRNA centered regulatory mechanism and identify novel biomarkers for the diagnosis and treatment of APA.

In the current study, bioinformatics analyses for the expression profile of APA compared with AAG were conducted aiming at identifying novel biomarkers and therapeutic targets of APA. First, bioinformatics analyses were performed based on the expression profiles of GSE60044, GSE64957, and GSE101894 datasets to identify differentially expressed genes (DEGs). Second, function and pathway enrichment analyses and protein–protein interaction (PPI) network analyses were conducted based on DEGs to identify essential biological processes and pathways contributing to APA. Third, ceRNA regulatory network was constructed to uncover hub genes that involved in the development of APA based on the differentially expressed lncRNAs, mRNAs, and the predicted miRNAs binding with differentially expressed lncRNAs.

## 2 MATERIALS AND METHODS

### 2.1 Data resource

Gene expression data of APA were searched in the Gene Expression Omnibus (GEO) database, The Cancer Genome Atlas (TCGA) database, the ArrayExpress database, InSilicoDB, and PubMed. Detailed searching and cohort processing strategies were documented in previous works.^16^, ^17^ This study analysed datasets that met the following inclusion criteria: (a) sequencing results obtained from human adrenal tissues; (b) transcriptome data from both APA and AAG were available; and (c) transcriptome data derived from expression profile microarray or high throughput RNA‐sequencing. After datasets filtering, three gene expression microarray datasets (GSE60044, GSE64957, and GSE101894) were included for further analyses. GSE60044 dataset was produced on a GPL14450 Agilent‐028004 SurePrint G3 Human GE 8x60K Microarray platform, GSE64957 was produced on a GPL10739 [HuGene‐1_0‐st] Affymetrix Human Gene 1.0 ST Array platform, and GSE101894 was produced on a GPL19748 Agilent‐038314 CBC Homo sapiens lncRNA+mRNA microarray V2.0 platform. Finally, 23 APA samples and 36 AAG samples were included for bioinformatic analyses.

### 2.2 Data preprocessing and DEG analyses

The getGEO function of the GEOquery package were applied to annotate the probe sets. Linear models for microarray data algorithms (LIMMA) package was used to normalize gene expression data and identify differentially expressed genes (DEGs) between APA and AAG samples.^18^ Genes with adjusted *p* value <0.05 and |log2(fold change)| >1.5 were defined as statistically significant DEGs. *p* values were adjusted using the Benjamini–Hochberg false discovery rate (FDR). The ggpubr R package was used to draw volcano plots for DEGs and the ComplexHeatmap R package was applied to draw heatmaps based on the top 50 DEGs.

### 2.3 Robust rank aggregation (RRA) analysis

Differentially expressed genes (DEGs) obtained from the three datasets were combined and filtered by the robust rank aggregation (RRA) R package.^19^ DEGs in all three datasets were ranked in ascending and descending order respectively according to the |log2(fold change)|, and then, the genes were re‐ranked by RRA according to the significance score. Significance score < 0.05 was regarded as the significance threshold. Further, ComplexHeatmap R package was applied to visualize the top 40 of the upregulated and downregulated significant robust DEGs.^20^

### 2.4 Gene ontology (GO) and kyoto encyclopedia of genes and genomes (KEGG) pathway analyses

ClusterProfiler R package was adopted to conduct the GO enrichment composed of biological process (BP), cellular components (CC), molecular function (MF),^21^ and KEGG pathway analysis^22^ to identify the biological functions and pathways with the significant robust DEGs. *p* value <0.05 and *q* value <0.05 were set as the thresholds for GO enrichment terms, while *p* value <0.05 and *q* value <0.2 were set as the significance cutoff criterion for KEGG pathway enrichment.

### 2.5 Protein–protein interaction (PPI) network

To uncover potential associations between DEGs, functional PPI networks were constructed by the Search Tool for the Retrieval of Interacting Genes STRING (http://www.string‐db.org). The Cytoscape software (v3.7.2) and Molecular Complex Detection (MCODE) (version 1.6.1) plugin were adopted to visualize the PPI network downloaded from STRING tool. We regarded modules with MCODE score ≥ 4 and nodes ≥5 as significant modules. All genes in the significant modules were considered to be the hub genes of this study.

### 2.6 Competing endogenous RNA (ceRNA) regulatory network construction and hub genes identification

To construct ceRNA network, we first identified differentially expressed lncRNAs from the GSE101894 dataset and then predicted the potential target miRNAs binding with the differentially expressed lncRNAs using the miRcode network tool (http://www.mircode.org/) based on the highly conserved miRNA family information provided by of the miRcode database.^23^ After obtaining the miRNAs bound to differentially expressed lncRNAs, these target miRNAs were further used to predict the mRNA bounding with them. The miRWalk network tool (http://miRWalk.umm.uni‐heidelberg.de/) was applied to conduct the miRNA and mRNA prediction based on the comparison of the annotation files of the miRDB, miRTarBase, and TargetScan databases with the criterion for the matching database number ≥2 and the score ≥0.9.^24^ The final hub genes of this study were identified by determining the overlapping genes of the hub genes derived from PPI and target mRNAs obtained from lncRNA‐miRNA‐mRNA predicting approach. The final hub genes were used to construct the ceRNA network. The ceRNA network was visualized by the Cytoscape (version 3.7.2).

## 3 RESULTS

### 3.1 DEGs between APA and AAG samples

**FIGURE 1:**
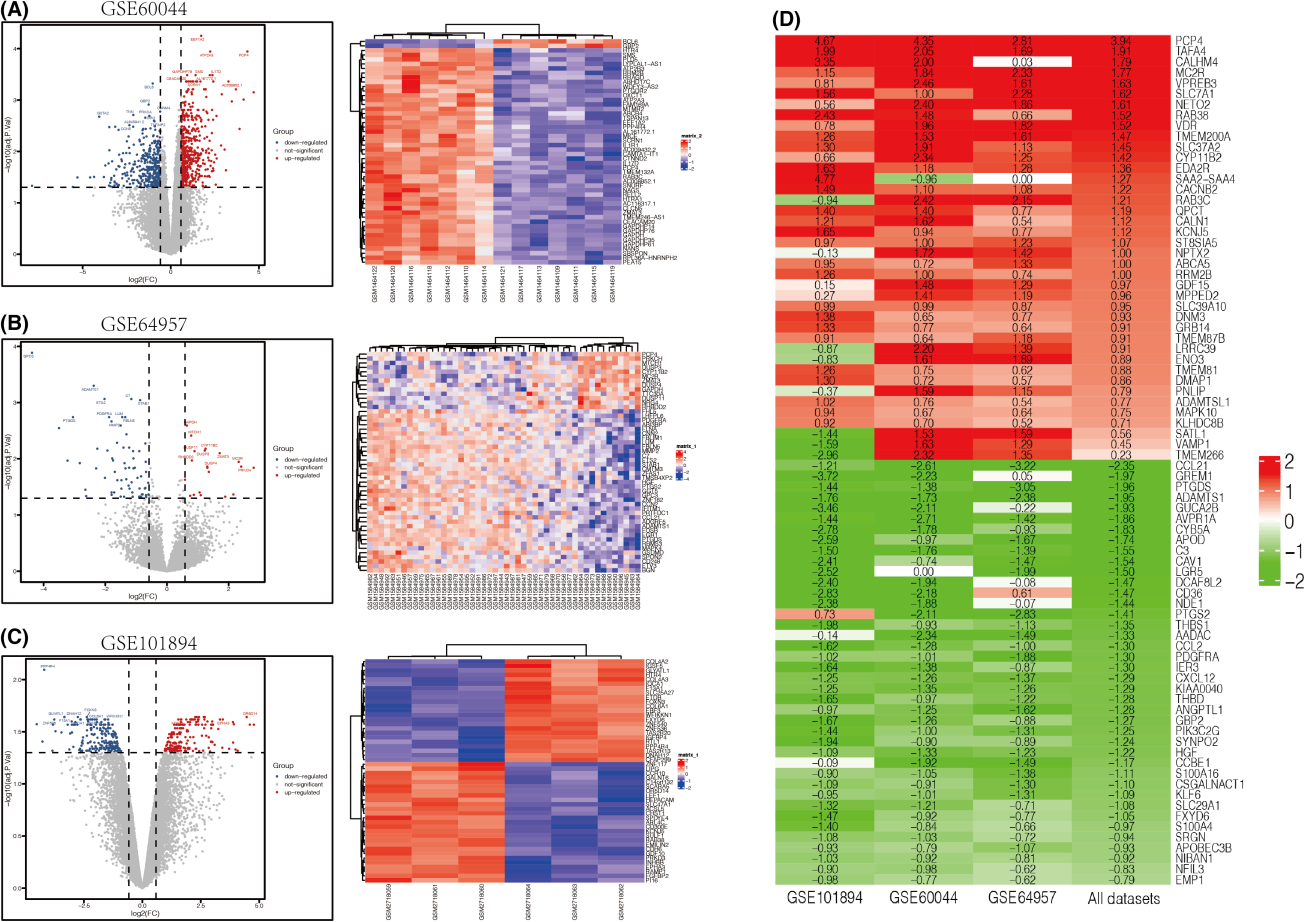
Differentially expressed genes (DEGs) in the three datasets. (A) Volcano plot of DEGs (left side) and heatmap (right side) of top 50 DEGs from GSE60044. A total of 899 DEGs were identified, including 532 upregulated genes (the red dots) and 367 downregulated genes (the blue dots). (B) Volcano plot of DEGs and heatmap of top DEGs from GSE64957. A total of 87 DEGs were identified, including 20 upregulated genes and 57 downregulated genes. (C) Volcano plot of DEGs and heatmap of top 50 DEGs from GSE101894. A total of 395 DEGs were identified, including 182 upregulated genes and 213 downregulated genes. (D) Integration of DEGs in all three datasets. DEGs were ranked in ascending and descending order respectively according to the |log2(fold change)|. ComplexHeatmap R package was applied to visualize the top 40 upregulated (red gradient) and downregulated (green gradient) integrated DEGs identified from robust rank aggregation analysis

Datasets were screened and filtered according to the inclusion and exclusion criteria. Three microarray datasets of APA and AAG samples were selected for further analyses. The GSE60044 included data of six APA and six AAG samples; the GSE64957 included data of 14 APA and 27 AAG samples; and the GSE101894 included data of three APA and threee AAG samples. Finally, 23 APA and 36 AAG samples were included. All datasets were normalized (Figure S1A,B,D,E,G,H) and subjected to PCA **(**Figure S1C,F,I**)**. We totally identified 899 DEGs in GSE60044, including 532 upregulated genes and 367 downregulated genes; 87 DEGs in GSE64957, including 20 upregulated genes and 67 downregulated genes; and 395 DEGs in GSE101894 dataset, including 182 upregulated genes and 213 downregulated genes. DEGs between APA and AAG in the three datasets were visualized by volcano plots. Heatmaps containing the top 50 most changed DEGs were drawn for the three datasets (Figure 1A–C).

### 3.2 RRA method identifies robust DEGs

Robust rank aggregation (RRA) approach was applied to integrate the three GSE datasets. On the basis of the results of the RRA analysis with the significance score <0.05, 3038 significant DEGs, including 1526 upregulated genes and 1512 downregulated genes were identified. Purkinje Cell Protein 4 (PCP4) ranked first among the upregulated genes (*p* = 1.33E‐12, adjusted *p* = 3.48E‐8), while ADAM Metallopeptidase with Thrombospondin Type 1 Motif 1 (ADAMTS1; *p* = 1.53E‐6, adjusted *p* = 0.04) was the most significant downregulated gene. The top 40 most significantly upregulated and downregulated genes in APA were visualized by a heatmap (Figure 1D **)**. Some of the significant DEGs have been well established as the pathogenic genes of aldosteronism, such as KCNJ5, CYP11B2, and CACANA1D. The role of the top up regulated gene PCP4 has not been investigated in aldosteronism. PCP4 functions as a modulator of calcium‐binding by calmodulin and plays a key role in Ca^2+^‐dependent signal transduction pathways.^25^ Given Ca^2+^ channel abnormality is a central pathogenic mechanism of aldosteronism, PCP4 may be a potential pathogenic gene of APA. The most significant downregulated gene ADAMTS1 is associated with inflammatory processes and has anti‐angiogenic activity.^26^ Thus, downregulation of ADAMTS1 may promote the angiogenic processes and contribute to the genesis of APA.

### 3.3 Functional enrichment analysis of integrated DEGs

**FIGURE 2:**
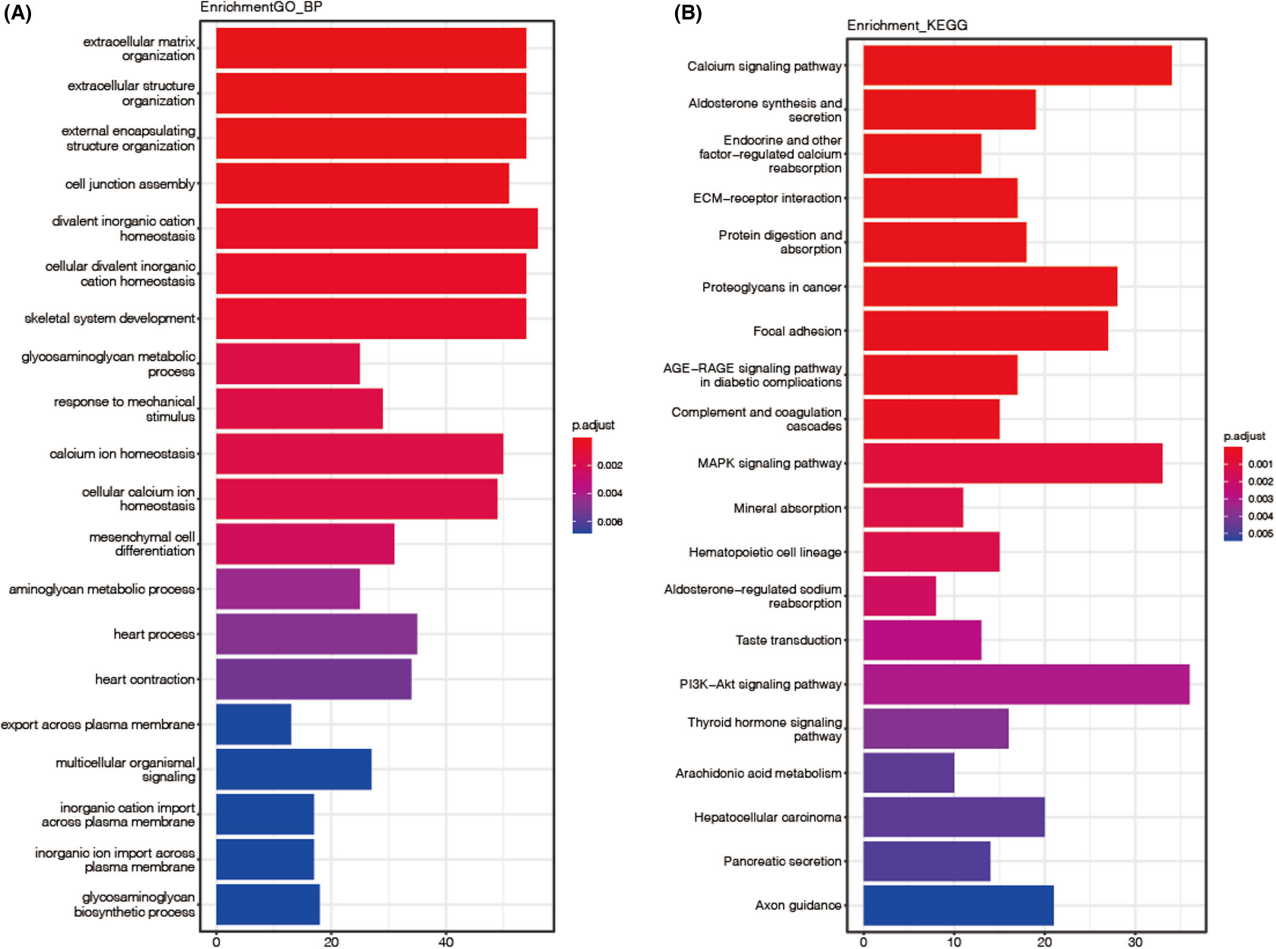
Enrichment analysis. (A and B) gene ontology (GO) (A) and kyoto encyclopedia of genes and genomes (KEGG) (B) enrichment analysis of all the integrated DEGs from the three GSE dataset. *p* value <0.05 and *q* value <0.05 were set as the thresholds for GO enrichment terms, while *p* value <0.05 and *q* value <0.2 were set as the significance cutoff criterion for KEGG pathway enrichment. Top 20 significant terms are shown

To identify predominant biological processes involved in the development of APA, we performed GO and KEGG enrichment analyses based on the significant DEGs. 79 significant GO terms were identified according to the criteria *p* < 0.05 and *q* < 0.05. The top ranked terms are mainly involved in the extracellular matrix and ion homeostasis, such as extracellular matrix organization (GeneRatio = 54/1036; *p* adjust = 1.41E‐6), cell junction assembly (GeneRatio = 51/1036; *p* adjust = 2.24E‐6), divalent inorganic cation homeostasis (GeneRatio = 56/1036; *p* adjust = 4.18E‐4), and Ca^2+^ homeostasis (GeneRatio = 50/1036; *p* adjust = 1.48E‐3). The top 20 GO terms are shown in Figure 2A.

In terms of KEGG, 22 significant KEGG pathways were uncovered. The high‐ranking pathways are involved in two major aspects, one is the Ca^2+^ signalling and aldosterone synthesis and secretion, such as Ca^2+^ signalling pathway (GeneRatio = 34/513; *p* adjust = 7.12E‐6) and aldosterone synthesis and secretion (GeneRatio = 19/513; *p* adjust = 9.75E‐6). The other one is pathways related to signal transduction from cell membrane to nucleus, such as MAPK signalling pathway (GeneRatio = 19/513; *p* adjust = 9.75E‐6) and PI3K − Akt signalling pathway (GeneRatio = 36/513; *p* adjust = 3.10E‐3). The top 20 KEGG pathways are shown in Figure 2B.

### 3.4 PPI Network identifies hub genes and GO enrichment analysis

**FIGURE 3:**
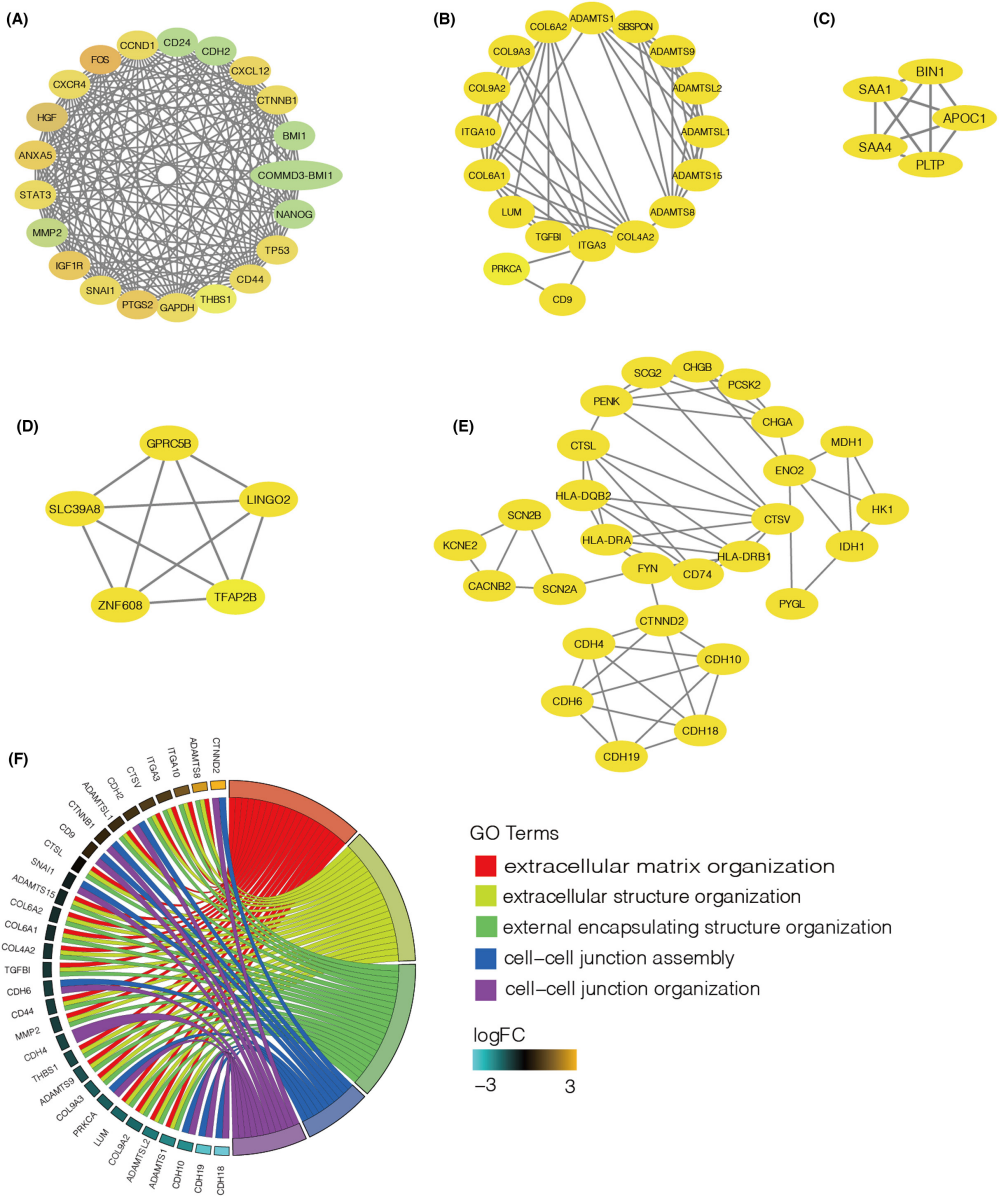
The protein–protein interaction (PPI) network of the integrated robust differentially expressed genes (DEGs). (A–E) Top 5 modules identified through MCODE in Cytoscape software. The size of the circle is positively correlated with the MCODE score and red represent the highest MCODE score while blue represent the lowest MCODE score. (F) GO enrichment analysis of the 68 hub genes identified in PPI network. logFC, log2 fold change

To screen for the genes that play essential roles in APA, the DEGs were further analysed using the STRING database to construct a PPI network and identify modules through the built‐in MCODE of Cytoscape software. Significant DEGs were uploaded to STRING and five significant modules were selected. The rank first module contains 21 nodes and 176 edges **(**Figure 3A **)**. To avoid one‐side data interpretation, we also included four other modules with score higher than 5.0 (Figure 3B–E **)**. The four modules contain 18 nodes/306 edges, 5 nodes/53 edges, 5 nodes/43 edges, and 27 nodes/378 edges, respectively. All genes in five modules are regarded as hub genes. As a result, 70 hub genes were revealed by PPI network analyses. GO function annotation of these hub genes were conducted. We found 68 potential genes enriched in 228 terms. The top ranked terms were mainly focused on the extracellular matrix related processes (Figure 3F).

### 3.5 CeRNA network construction

**FIGURE 4:**
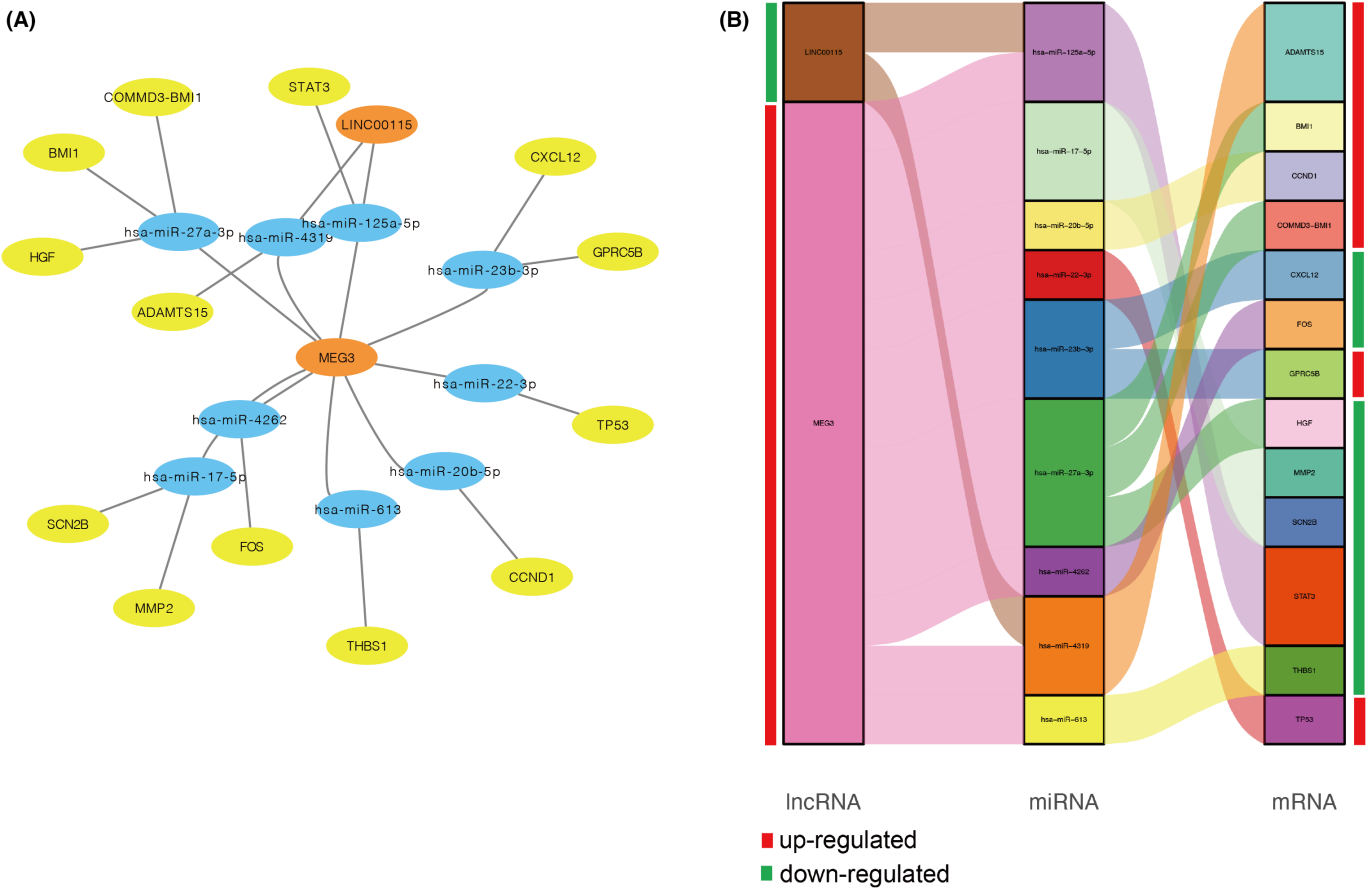
The potential competing endogenous RNA (ceRNA) network in aldosterone‐producing adenoma (APA). CeRNA regulatory network was constructed to uncover hub genes of APA based on the differentially expressed lncRNAs, mRNAs, and the predicted miRNAs binding with differentially expressed lncRNAs. The network included 2 lncRNAs, 9 miRNAs, and 13 mRNAs. The ceRNA network was visualized by the Cytoscape (A) and Sankey plot (B). The orange circle represents lncRNAs, the blue circle represents miRNAs, and the yellow circle represents mRNAs. The red strip represents upregulated genes, and the green strip represents downregulated genes

We identified 18 significantly differentially expressed lncRNAs from the GSE101894 dataset. Using the miRcode network tool, we obtained 192 potential target miRNAs binding with the differentially expressed lncRNAs. Further, the 192 target miRNAs were used to predict the mRNAs bounding with them by the miRWalk network tool. A total of 2795 mRNAs were identified to bind with these miRNAs. Finally, we took the intersection of the 68 potential genes identified by PPI analyses and the 2795 mRNAs predicted from miRNAs. As a result, a total of 13 hub mRNAs were uncovered. Consequently, the final ceRNA network included 2 lncRNAs, 9 miRNAs, and 13 mRNAs. Then, we visualized the ceRNA network by the Cytoscape (Figure 4). Of the 2 lncRNAs and 13 mRNAs in the ceRNA network, 7 were upregulated in APA, including MEG3, CCND1, TP53, GPRC5B, BMI1, COMMD3‐BMI1, and ADAMTS15. The rest were downregulated in APA, including LINC00115, STAT3, MMP2, SCN2B, CXCL12, HGF, FOS, and THBS1.

## 4 DISCUSSION

Aldosterone‐producing adenoma (APA) is a complex endocrine disease whose pathogenesis has not been fully illuminated. Genes related with ion channels and aldosterone synthesis are crucial regulators of occurrence and development of APA.^5^ Although many expression profile analyses have been performed, no study have investigated the roles of ceRNA in the pathogenesis of APA, especially lncRNAs acting as ceRNAs participating in the regulation of gene expression. This is the first study to perform ceRNA regulatory network analyses to identify hub genes that contribute to the pathogenesis of APA. Overall, the findings of this systematic microarray analysis may help us to identify novel biomarkers and therapeutic targets of APA.

Previous studies have revealed that mutations affecting ion channels (Ca^2+^, K^+^, Na + K+ ATPase) identified in PA patients can lead to elevated intracellular Ca^2+^ concentrations and autonomous aldosterone secretion. Mechanically, mutations shift the voltage dependence of the channel to a more negative potential, or cause delayed voltage‐dependent inactivation of the channel, leading to increased calcium entry into the cell, which subsequently activates Ca^2+^ signalling and aldosterone over production.^27^, ^28^ The bioinformatics analysis conducted in this study totally identified 1526 upregulated and 1512 downregulated DEGs. Functional enrichment analyses revealed that DEGs were predominantly enriched in Ca^2+^ ion homeostasis, aldosterone synthesis and secretion, as well as tumorigenesis. Therefore, the results of this study verified previous findings, suggesting the results obtained from the current study are of high reliability.

Although the roles of lncRNA and ceRNA regulatory mechanism have been demonstrated in tumorigenesis diseases,^5^ no study has ever investigated the role of ceRNA network in APA. Thus, there is an urgent need to explore the ceRNA regulatory mechanism in APA. In this study, we first identified the differentially expressed lncRNAs in APA, and then used these lncRNAs to predict target miRNAs binding with them. Further we analysed mRNAs that bind with the miRNAs. Finally, the intersection of the predicted mRNAs and the potential mRNAs obtained from PPI were regarded as the hub mRNAs of the ceRNA network. The final ceRNA network included 2 lncRNAs, 9 miRNAs, and 13 mRNAs. We uncovered several lncRNA‐miRNA‐mRNA interaction links that may be involved in the development and biological characteristics regulation of APA, such as MEG3‐has‐miR‐22‐3p‐TP53, MEG3‐has‐miR‐4319‐ADAMTS15, LINC00115‐has‐miR‐125a‐5p‐STAT3, and LINC00115‐has‐miR‐20b‐5p‐CCND1.

LncRNA MEG3 is a lncRNA tumour suppressor. Its expression is lost in multiple cancer cell lines and could inhibit tumour cell proliferation in vitro^29^ as well as enhance angiogenesis processes in vivo.^30^ Additionally, MEG3 can also interact with the tumour suppressor TP53 and regulate TP53 target gene expression.^31^ In the current study, we found MEG3‐has‐miR‐22‐3p‐TP53 may be an essential ceRNA interaction link for APA. In this pathway, the expression level of tumour suppressor lncRNA MEG3 and the tumour suppressor gene TP53 were significantly upregulated. This might be one of the reasons why APA has slower speed of growth and proliferation, smaller tumour size, and noninvasive biological behaviour compared with malignant tumours. MEG3‐has‐miR‐4319‐ADAMTS15 pathway is another ceRNA interaction link uncovered to participate in APA development and biological characteristics regulation. ADAMTS15 encodes a member of the ADAMTS (a disintegrin and metalloproteinase with thrombospondin motifs) protein family and has been shown to function as a tumour suppressor by decreasing cell migration and invasion.^32^ Similar to TP53, the tumour suppressor ADAMTS15 was also significantly upregulated in APA patients, which further confirmed the phenomenon that tumour suppressor genes were upregulated in APA. CCND1 is another hub mRNA identified in the MEG3 based ceRNA link. It encodes cyclin D1, a highly conserved cyclin family member. Mutations, amplification, and overexpression of this gene could alter cell cycle progression.^33^ Thus, we speculate that the activation of CCND1 may promote APA formation by directly altering the cell cycle progression.

As the central mechanism leading to aldosterone over production in APA is somatic mutations of ion channel genes that lead to increased intracellular Ca^2+^ level, enhanced aldosterone synthase expression, and excessive aldosterone production. Thus, the expression level changes in ion channel genes may be of great significance to APA. In the current study, MEG3‐has‐miR‐17‐5p‐SCN2B was identified to be one of the essential ceRNA interaction links of APA. SCN2B encodes the beta 2 subunit of the type II voltage‐gated sodium channel and defects in SCN2B are related to Brugada Syndrome, atrial fibrillation, and sudden infant death syndrome.^34^ However, the role of SCN2B in APA has not been investigated. In this study, the expression level of SCN2B is downregulated in APA samples. This may lead to the depolarization of cell membrane. The increased Ca^2+^ level of adrenal glomerulosa cells may finally promote the synthesis of aldosterone.

LINC00115 is another lncRNA revealed by the ceRNA network analysis. Genome wide association studies (GWASs) showed that LINC00115 polymorphism is associated with multiple types of tumours^35^, ^36^, ^37^, ^38^ Our results showed that LINC00115‐has‐miR‐125a‐5p‐STAT3 may serve as an important ceRNA interaction link for APA. STAT3 mediates the expression of a variety of genes in response to cell stimuli, and thus plays a key role in cell growth and apoptosis as an oncogenic gene. Kiyotaka Itcho et al reported that STAT3 influence aldosterone production by negatively regulate the Ang 1–7 mediated aldosterone production processes in human adrenal cells.^39^ In the LINC00115‐has‐miR‐125a‐5p‐STAT3 pathway, the expression of LINC00115 and oncogenic gene STAT3 were significantly downregulated, which reversely illustrated the benign biological behaviour of APA. Interestingly, LINC00115 polymorphism was also documented by the GWAS to correlate with systolic blood pressure (SBP), however, the potential mechanism of LINC00115 affecting SBP has not been illustrated. As PA is one of the most prevalent forms of secondary hypertension, the reason for the correlation between LINC00115 and SBP might be that it involved in the development of APA.

The ceRNA interaction pathways identified by the current study provide new insights into the pathogenesis of APA. The construction of ceRNA network offers potential therapeutic targets and diagnostic biomarkers for APA. Further in vitro and in vivo experiments are needed to confirm the functional roles of these lncRNA, mRNAs, and miRNAs. If their functions are confirmed experimentally, targeted intervention can be carried out for specific genes and related pathways, which may be expected to become new therapeutic approaches for APA, or even become an alternative strategy for invasive adrenalectomy.

This study has some advantages and limitations. One of the advantages is that this is the first study to explore the lncRNA‐centered regulatory mechanism by constructing ceRNA network on APA. Another advantage is that the systematic analysis uncovered 2 lncRNAs, 9 miRNAs, and 13 mRNAs that may be novel biomarkers for the diagnosis and treatment of APA. The limitations of this study lie in three aspects. First, APA tissue specimens were not obtained from patients to replicate the findings of this study. Second, in vitro and in vivo experiments are needed to confirm the functional roles and identify potential mechanisms of hub genes on APA. Third, only one dataset in the GEO database explored lncRNAs that contribute to APA, lacking sufficient transcriptome data may lead to compromised ability to identify differentially expressed lncRNAs.

## 5 CONCLUSIONS

This is the first study to explore the potential role of ceRNAs in APA via bioinformatics approach. CeRNA analyses identified MEG3 and LINC00115 as two central lncRNAs that may serve as primary regulators for APA. In addition, 9 miRNAs and 13 mRNAs were also uncovered to play essential roles in APA. Our findings provide new insights into the pathogenic mechanisms of APA and uncover potential diagnostic biomarkers and intervention targets.

## AUTHOR CONTRIBUTIONS

**Minghui Bao:** Conceptualization (lead); data curation (equal); formal analysis (equal); funding acquisition (lead); investigation (lead); methodology (lead); project administration (equal); resources (lead); software (equal); supervision (equal); validation (equal); visualization (lead); writing – original draft (lead); writing – review and editing (equal). **Haotong Li:** Conceptualization (supporting); data curation (equal); formal analysis (equal); software (supporting). **Jianping Li:** Conceptualization (equal); investigation (supporting); project administration (equal); supervision (lead); writing – review and editing (equal).

## CONFLICT OF INTEREST

The authors confirm that there are no conflicts of interest.

## DISCLOSURE

GEO is a public database. The patients involved in the database have obtained ethical approval. Users can download relevant data for free for research and publish relevant articles. Our study is based on open source data, so there are no ethical issues and other conflicts of interest.

## FUNDING STATEMENT

This study was supported by the National Natural Science Foundation of China (No.82000432) and the Peking University First Hospital Seed Foundation (No. 2020SF01).

## Supporting information


FigureS1

## Data Availability

Generated Statement: Publicly available datasets were analysed in this study. These data can be found from: https://www.ncbi.nlm.nih.gov/geo/.
