# Supplementary figures and images for: Identification of potential lncRNA‐miRNA‐mRNA regulatory network contributing to aldosterone‐producing adenoma
Source: J Cell Mol Med. 2022 Oct 27;26(22):5614–23. doi: 10.1111/jcmm.17586 (PMC9667512; doi:10.1111/jcmm.17586)

**A**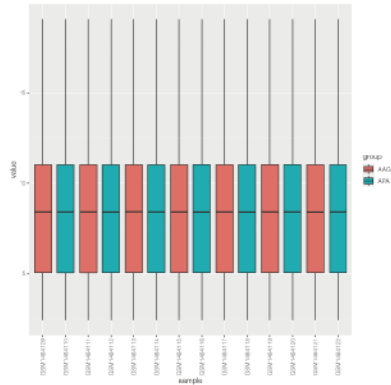**B**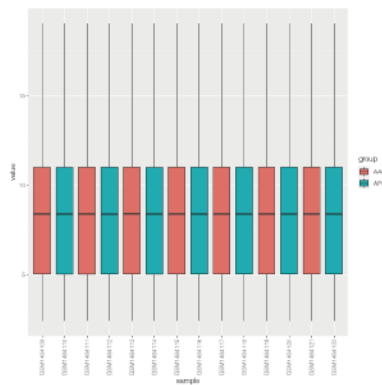**C**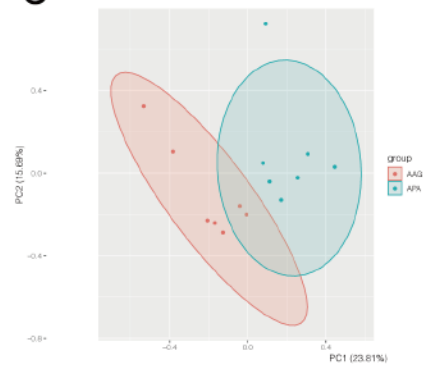**D**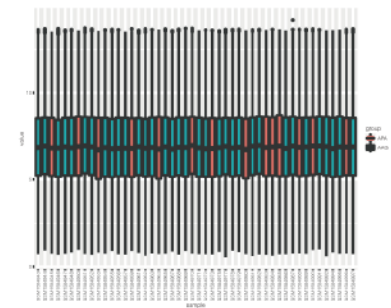**E**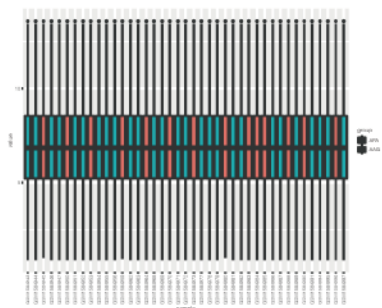**F**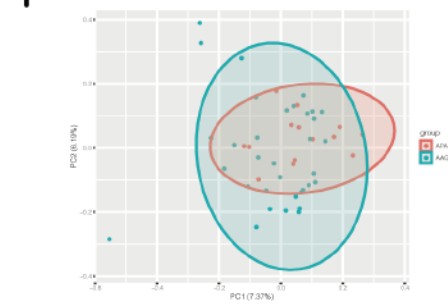**G**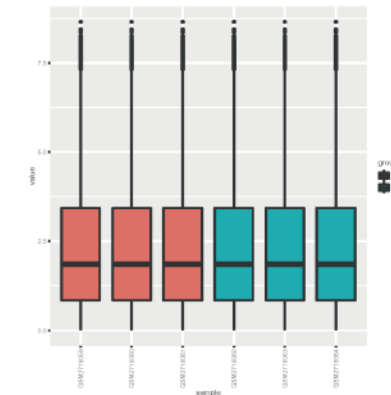**H**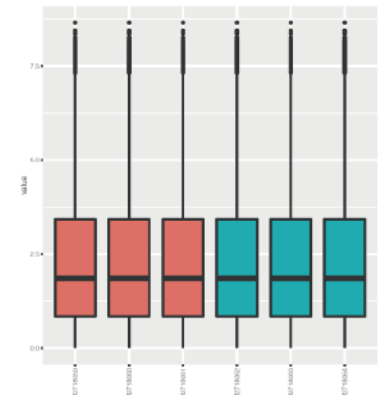**I**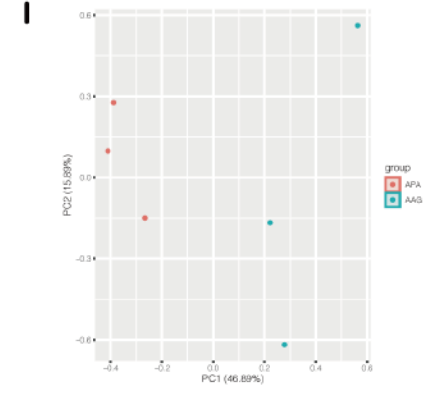

Supplement: Supplementary file 1 — FigureS1 [file JCMM-26-5614-s001.pdf]
